# Development of a miRNA‐based classifier for detection of colorectal cancer molecular subtypes

**DOI:** 10.1002/1878-0261.13210

**Published:** 2022-04-29

**Authors:** Ronja S. Adam, Dennis Poel, Leandro Ferreira Moreno, Joey M. A. Spronck, Tim R. de Back, Arezo Torang, Patricia M. Gomez Barila, Sanne ten Hoorn, Florian Markowetz, Xin Wang, Henk M. W. Verheul, Tineke E. Buffart, Louis Vermeulen

**Affiliations:** ^1^ Laboratory for Experimental Oncology and Radiobiology (LEXOR) Center for Experimental and Molecular Medicine (CEMM) Cancer Center Amsterdam and Amsterdam Gastroenterology and Metabolism Amsterdam University Medical Centers The Netherlands; ^2^ 26066 Oncode Institute Amsterdam The Netherlands; ^3^ Department of Medical Oncology Radboud University Medical Center Nijmegen The Netherlands; ^4^ Cancer Research UK Cambridge Institute University of Cambridge UK; ^5^ Department of Biomedical Sciences City University of Hong Kong Kowloon Tong Hong Kong; ^6^ Shenzhen Research Institute City University of Hong Kong Shenzhen China; ^7^ 26066 Department of Gastrointestinal Oncology Netherlands Cancer Institute Amsterdam The Netherlands

**Keywords:** colorectal cancer, consensus molecular subtypes, microRNA, miRNA

## Abstract

Previously, colorectal cancer (CRC) has been classified into four distinct molecular subtypes based on transcriptome data. These consensus molecular subtypes (CMSs) have implications for our understanding of tumor heterogeneity and the prognosis of patients. So far, this classification has been based on the use of messenger RNAs (mRNAs), although microRNAs (miRNAs) have also been shown to play a role in tumor heterogeneity and biological differences between CMSs. In contrast to mRNAs, miRNAs have a smaller size and increased stability, facilitating their detection. Therefore, we built a miRNA‐based CMS classifier by converting the existing mRNA‐based CMS classification using machine learning (training dataset of *n* = 271). The performance of this miRNA‐assigned CMS classifier (CMS‐miRaCl) was evaluated in several datasets, achieving an overall accuracy of ~ 0.72 (0.6329–0.7987) in the largest dataset (*n* = 158). To gain insight into the biological relevance of CMS‐miRaCl, we evaluated the most important features in the classifier. We found that miRNAs previously reported to be relevant in microsatellite‐instable CRCs or Wnt signaling were important features for CMS‐miRaCl. Following further studies to validate its robustness, this miRNA‐based alternative might simplify the implementation of CMS classification in clinical workflows.

## Graphical abstract

Consensus molecular subtypes (CMSs) capture heterogeneity of colorectal cancer based on mRNA expression. To retrieve CMS classification from miRNAs, we present a miRNA‐assigned CMS classifier (CMS‐miRaCl), which we validated using several datasets. Important features of this random forest model were linked to microsatellite instability or Wnt signaling. CMS‐miRaCl potentially facilitates CMS classification using miRNA data in clinical workflows and research settings.

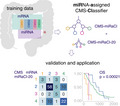

**Abbreviations**
CI: confidence interval
CMS: consensus molecular subtype
COAD: colon adenocarcinoma
CRC: colorectal cancer
dn: downregulated in
DPI: data processing inequality
EGAS1127: European Genome‐phenome Archive study EGAS00001001127
EMT: epithelial‐mesenchymal transition
Epith.: epithelial
log2FC: log2 fold change
Mesench.: mesenchymal
miR, miRNA: microRNA
miRaCl: microRNA‐assigned CMS classifier
mRNA: messenger RNA
OS: overall survival
*P*adj: adjusted *P*‐value
Prot.: protein
qPCR: quantitative polymerase chain reaction
READ: rectal adenocarcinoma
Resp.: response
RTN: regulatory transcriptional networks
Sign.: signaling
TCGA: The Cancer Genome Atlas
Trans.: transition
tSNE: t‐distributed stochastic neighbor embedding

## 1 Introduction

### 1.1 CMS based on mRNA and alternatives

To understand the intertumor heterogeneity of colorectal cancer (CRC), tumors have been classified into consensus molecular subtypes (CMSs), which reflect their molecular characteristics [1]. By studying transcriptomic features, we and others were able to characterize these four main disease subtypes with implications for clinical outcome, response to therapy, and fundamental disease mechanisms [1, 2, 3, 4, 5]. Since then, several studies have attempted to extract this classification from other data types, in order to widen the applicability to clinical and research contexts.

For example, the CMS classifier was adapted for NanoString gene panels for RNA from formalin‐fixed paraffin‐embedded tissue [6, 7], and transcriptome microarray data were used to build a qPCR‐based classifier for the most aggressive CMS, CMS4 [8]. Furthermore, the CMS classifier has been applied to analyze histology slides via a neural network‐based image analysis approach, imCMS, or via small panels of immunohistochemical stainings [9, 10, 11].

The profiling of other genomic data types demonstrated incomplete associations of CMSs with mutations, methylation, and miRNAs [1]. Since previous studies revealed that gene expression profiles of CMSs are partially regulated by miRNAs [1, 12], this study examined whether CMSs can be determined directly from miRNA expression levels.

### 1.2 miRNAs in cancer and CRC

miRNAs are small noncoding RNAs of 18–25 nucleotides with wide regulatory functions including initiating decay or blocking translation of specific target mRNAs in the cytoplasm. Moreover, as an adverse function, transcription‐activating interactions with promotor regions have been described for miRNAs that translocate from the cytoplasm into the nucleus. Since their target interaction typically requires a match of only 7–8 nucleotides to the 3′ untranslated region of the mRNA, which might allow for a mismatch, the range of potential targets is large. However, the effect size of a single interaction is usually low and depends on the expression levels of the target. Therefore, efficient regulation is often achieved by targeting multiple genes of a pathway and/or additive effects of commonly transcribed miRNA clusters or families [13].

miRNAs are relatively stable even in tissues of compromised quality [14]; thus, they are frequently investigated as biomarkers. A significant number of miRNAs have been found to be upregulated or downregulated in cancerous compared to normal colorectal tissue as reviewed by Pidíkova et al. [15]. Furthermore, the expression profiles of miRNAs seem to be more tissue‐specific than those of mRNAs [16]. There have been indications that miRNA–mRNA interactions might be context‐dependent and could even differ between molecular cancer subtypes [17, 18]. A comparison between CMSs, on the level of cell lines, suggested regulatory roles of miR‐194 (from the 192/194/215‐cluster) and the miR‐200 family [19]. The latter is critical in establishing and maintaining epithelial cell identity, and both are downregulated in CMS4 [12, 20].

### 1.3 Study setup

To investigate to which extent the miRNA transcriptome can separate the CMS classes we trained a random forest classifier using miRNA expression data for which standard CMS classes was available from paired mRNA data. During this supervised training of a miRNA‐assigned classifier, we gained additional insights into the regulation of CMSs through miRNAs. The testing performed in a different dataset showed an accuracy of 76–79% for samples with high prediction confidence.

## 2 Materials and methods

### 2.1 Experimental setup

We trained a random forest classifier using miRNA expression data from The Cancer Genome Atlas (TCGA) colon adenocarcinoma (COAD) dataset (271 samples) to identify CMS classes, which we obtained from paired mRNA data [21]. The classifier was tested using miRNA expression data from TCGA rectal adenocarcinoma (READ) compared with the standard CMS classes from paired mRNA data (158 samples) [21], and miRNA expression data with clinical data from a cohort of 126 primary samples of CRC with metastases, EGAS00001001127 (abbreviated as EGAS1127) [22, 23]. Furthermore, we tested the classifier on primary COAD samples, which were difficult to classify based on mRNA and had thus not been used for training (*n* = 169). COAD sample pairs from fresh frozen and formalin‐fixed, paraffin‐embedded (FFPE) tissue were used to test applicability to FFPE samples (*n* = 7). In addition, we examined primary samples of the datasets GSE29623 (*n* = 65) and GSE35834 (*n* = 31) concerning the generalization to microarray‐based data. Our scripts and the classifiers are publicly available via Github/rsmadam/CMS‐miRaCl.

### 2.2 Data retrieval and preprocessing

We retrieved COAD miRNA data using tcgabiolinks (2.14.1) [24]. We used only primary tumor samples from the first vial (sample/vial‐ID ‐01A). Isoforms were summarized as mean expression. To normalize the miRNA count data, we used variance stabilizing transformation from deseq2 (1.26.0) [25]. During principal component and t‐distributed stochastic neighbor embedding (tSNE) analyses using caret (6.0–85), we identified batch effects in the miRNA datasets from COAD related to tissue source sites, and we used limma (3.42.2) to remove them (Fig. S1A,B) [26, 27]. For COAD (*n* = 445), we obtained the CMS annotation from our previous work [1]. In addition, we generated the mRNA‐based CMS labels via the R package cmsclassifier (1.0.0), applying the Random Forest classifier to the COAD mRNA data. For this, we obtained COAD mRNA data from tcgabiolinks as RSEM normalized counts, which we log‐transformed and applied batch effect removal concerning the different platforms GA/HiSeq. Only labels that were reclassified concordant to our previous classification and had a *P*‐value < 0.05 were considered as robust (*n* = 276). COAD samples were thus excluded from the training dataset if they were nonclassifiable, e.g., due to the presence of intermediate subtypes or intratumor heterogeneity. We removed outlier samples (*n* = 5) using the Tukey's mild outlier definition. Features with low variance < 0.5 or high correlations > 0.75 were removed using caret (6.0–85) [26]. The remaining 381 miRNAs were considered suitable for the classifier training.

After obtaining the READ miRNA data from tcgabiolinks, we proceeded similarly, performing an independent variance stabilizing transformation and removal of batch effects related to tissue source sites. We used only primary tumor samples from the first vial (sample/vial‐ID ‐01A) (*n* = 158) and did not remove outliers. We performed log transformation of READ RSEM counts and removed batch effects from sequencing platforms to obtain CMS class labels.

EGAS1127 data was obtained from fresh frozen tissue samples of metastasized CRC as described previously [22, 23]. It comprised 126 primary tumor samples, of which 38 had at least one matched metastasis sample. We used these metastatic samples purely for creating a comparison between metastases and primary tumors (Fig. 5D). The 38 primary samples matched to 46 metastatic samples as follows: 30 primaries matched to 30 single metastatic samples, one primary matched to a single metastasis plus a local recurrence, and seven primaries matched to every two metastatic samples (corresponding to two different sites). Raw data underwent summarization of isoforms and variance stabilizing transformation and showed no obvious batch effects.

For the microarray‐based datasets GSE29623 and GSE35834, we obtained mRNA and miRNA data from Gene Expression Omnibus [28, 29]. We excluded two outliers in GSE29623 based on the Tukey's mild outlier definition. In the miRNA data, isoforms were summarized and each feature was scaled by division by their standard deviation. In the mRNA data, Affymetrix identifiers were translated to Entrez identifiers using biomaRt [30] before applying the CMS classifier.

### 2.3 Differential expression analysis

deseq2 (1.26.0) was applied for variance stabilizing transformation and differential expression analysis of raw read counts for both miRNA and mRNA data from COAD [25]. The results were annotated with org.Hs.eg.db (3.10.0) and plotted using complexheatmap (2.2.0) [31].

### 2.4 Classifier training

We used the caret package (6.0–85) for classifier development [26], with the goal to predict CMS labels from miRNA data. For this supervised classifier training, we combined the COAD miRNA data with the mRNA‐based CMS labels. To identify the optimal classifier algorithm, we compared accuracy and Kappa (accuracy with correction for random predictions) in random forests and support vector machines, each maximizing either accuracy or Kappa. After deciding for the ranger implementation of a random forest classifier, the optimal parameters were determined in a grid search [32]: We ran 100× repeated 10‐fold cross‐validations, using downsampling to balance the class composition. The importance of features was determined based on the Gini importance, which is equivalent to the mean decrease in Gini impurity. This indicates the pureness of the sample classes after separating samples based on this feature—compared with randomly picking a class label (respecting the class distribution). The values were scaled to a maximum of 100. To identify the average Gini importance of features and the accuracy on the training data, we created 100 leave‐out partitions of 20% and reran 100 times a 10× repeated 10‐fold cross‐validation to optimize parameters in the 80% subset before training the classifier (maximizing Kappa) on the 80% subset. The Gini importance showed a steep decrease within the 10 most important features and a minimal decrease after the 20th rank on average (Fig. S2A). With the optimized parameters (number of features to try (mtry) = 25 for miRaCl or mtry = 2 for miRaCl‐20, number of nodes = 10, number of trees = 2000), we trained a classifier (maximizing Kappa) on the entire COAD training dataset (*n* = 271) with 381 miRNAs (miRaCl) or 20 most important miRNAs (miRaCl‐20). We also explored further decreasing the number of features, but the accuracy decreased when we kept only 12 or 10 of the most important features (Fig. S2B).

### 2.5 Classifier evaluation

The classifier was applied to READ and EGAS1127 datasets to evaluate performance on two completely unrelated datasets. Furthermore, we applied the classifier to samples from COAD, which were not robustly classified by the mRNA‐based method and therefore excluded from training. Similarly, in READ, we required a robust mRNA‐based CMS label for the test set. Samples from READ, which were not robustly classified by the mRNA‐based method, were evaluated separately. The output of class probabilities was used to estimate the prediction confidence as the absolute difference between first and second highest class probabilities since similar probabilities between the predicted first class and second class indicate low confidence in the class decision. Prediction comparisons were plotted using pheatmap (1.0.12) for confusion matrices or ggalluvial (0.12.2) for alluvial plots [33]. The correlation was tested using the Spearman method in R package ggpubr (0.2.5) and plotted in ggplot2 (3.2.1) [34]. The ability to predict CMS based on 20 miRNAs was validated using two independent microarray‐based datasets, of which the larger GSE29623 was used to retrain the classifier to allow the input of scaled microarray data, and the smaller dataset GSE35834 was used to test the accuracy as reported. The clinical relevance of the miRNA‐based CMS classification for the overall survival (OS) was tested in the EGAS1127 dataset using a Cox proportional hazards regression model for association with the most informative miRNAs and a Kaplan–Meier analysis with a log‐rank test in R package survival (3.1–8) [35]. OS was defined as the time between the start of first‐line treatment until death from any cause and OS data. OS information was available for 82 out of 126 patients from the EGAS1127 dataset. For validation, the hazard ratios were also calculated in TCGA CRC (COAD+READ) samples with available OS data (*n* = 594), and a subset of patients with Stage IV (*n* = 87).

### 2.6 Network analysis

Regulatory transcriptional networks (RTN) were constructed using rtn package (2.10.1) and visualized using reder (1.34.0) [36, 37, 38]. The RTN method is based on the aracne algorithm [39], thus it identifies potential interactions, i.e., co‐expression, from expression data by measuring mutual information. The use of the mutual information criterion allows for capturing of interactions that are nonlinear, in contrast to using a correlation coefficient. Before the network is inferred, the identified interactions are reduced to the most significant ones by permutation analysis and bootstrapping and testing for data processing inequality (DPI) to prefer direct interactions over indirect interactions. From dataset COAD, we used the most differentially expressed mRNAs (absolute log2 fold change |log2FC| > 0.85, adjusted *P*‐value (*P*adj) < 0.001, at most 200) and the most differentially expressed miRNAs (|log2FC| > 0.71 and *P*adj < 0.05) per CMS based on Wald statistic with Benjamini–Hochberg corrected *P*‐values, related to previous proceedings [12]. The expression data of these genes (RSEM/RPM‐normalized scaled read counts) were evaluated for mutual information with the subgroup of either upregulated or downregulated miRNAs with *P*adj < 0.001 in each CMS, respectively, for the network inference. Benjamini–Hochberg method was applied to account for multiple testing, the unstable or redundant interactions were filtered via bootstrap and DPI filter. For visualization, we added the log2FC from deseq2 as a node color and the feature importance as node sizes, feature names were reduced to those present in miRaCl and miRaCl‐20.

### 2.7 Pathway analysis

We identified potential targets of the 20 most important miRNAs from the databases miRDB, miRTargetScan, and miRbase [40, 41, 42]. We ranked the predicted target genes based on the (experimental) support type and the number of databases they were listed in. To exclude low evidence targets and make results between miRNAs more comparable, we considered only genes predicted by at least two databases and a maximum of 200 predicted targets per miRNA. To analyze which pathways these target gene candidates were involved in, we tested for overlap with Hallmark gene sets with one‐sided hypergeometric tests equivalent to the Fisher's exact test using limma [27]. Hallmark gene sets are curated gene sets with experimental evidence and a maximum size of 200 [43]. Of note, this procedure may still produce false‐positive predictions. To narrow down the predicted pathways towards potential roles of the miRNAs in the phenotypes of the CMS, we performed an alternative analysis, where we first intersected the predicted targets with genes that are differentially expressed between the CMS. For each CMS we used the 200 most downregulated genes to obtain gene sets of the same size and facilitate comparability between CMSs (Table S1). We then performed a pathway overlap analysis for each CMS individually and showed the miRNA‐pathway overlap predictions for each CMS with the lowest *P*‐value in the one‐sided hypergeometric test.

## 3 Results

### 3.1 Experimental setup and dataset description

**Fig. 1:**
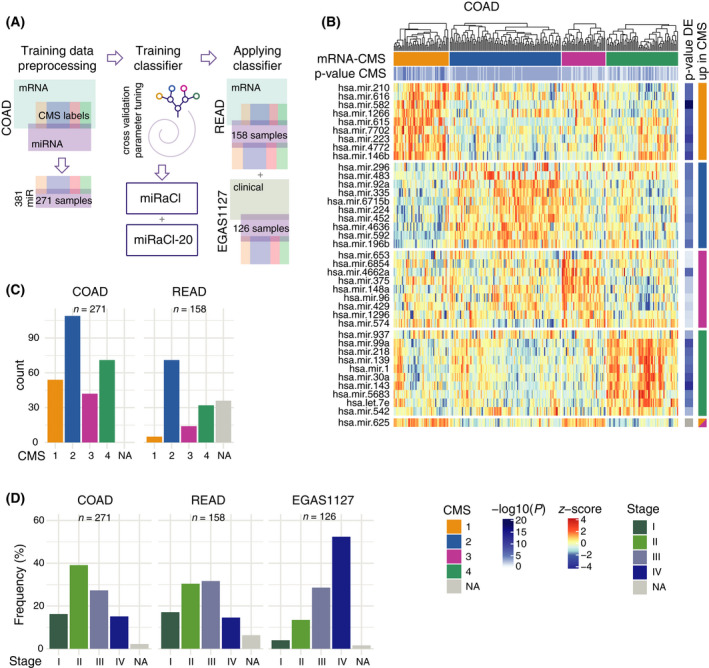
Experimental setup and dataset description. (A) Schematic representation of the workflow used to train and validate the consensus molecular subtype miRNA‐assigned classifier (CMS‐miRaCl). (B) Heatmap of the 10 most significantly differentially expressed miRNAs in the colon adenocarcinoma (COAD) training dataset (*n* = 271) per CMS class, showing read counts after preprocessing and scaling to *z*‐scores. On top, the mRNA‐based CMS classification and *P*‐value from CMS classifier are depicted, and on the right, the Benjamini–Hochberg adjusted *P*‐value from Wald statistics and the mRNA‐based class in which the miRNA was significantly upregulated. (C) Counts of samples' mRNA‐based CMS classes in the COAD training dataset (*n* = 271) and the rectal adenocarcinoma (READ) test dataset (*n* = 158). (D) Frequencies of samples' pathology‐based stage in training and test datasets.

We used the COAD miRNA dataset with paired mRNA‐based CMS labels to train a miRNA‐assigned CMS classifier, CMS‐miRaCl, and a parsimonious version with only 20 features, miRaCl‐20. Its performance was tested primarily on two independent datasets, READ and EGAS1127 (Fig. 1A). In the training dataset, it was first tested whether there were significantly differentially expressed miRNAs between the CMSs (Fig. 1B). Of note, miR‐625 was significantly upregulated in CMS1 vs. other CMSs and in CMS3 vs. other CMSs and, respectively, downregulated significantly in CMS2 and CMS4.

Both datasets with previously determined CMS labels, COAD and READ, were composed of all four CMSs, with CMS2 representing the largest class (Fig. 1C). The tSNE analysis resulted in clustering of the mRNA‐based CMS subtypes in the COAD miRNA dataset, whereas the classes separated less clearly in the READ dataset. The clinical characteristics of the examined datasets exhibit differences regarding the composition of stages (Fig. 1D, Fig. S1E). In the EGAS1127 dataset, 52% of the samples was of advanced tumor stage IV. Additionally, other primary tumors in the EGAS1127 dataset developed metachronous metastases.

### 3.2 Classifier training and performance evaluation

**Fig. 2:**
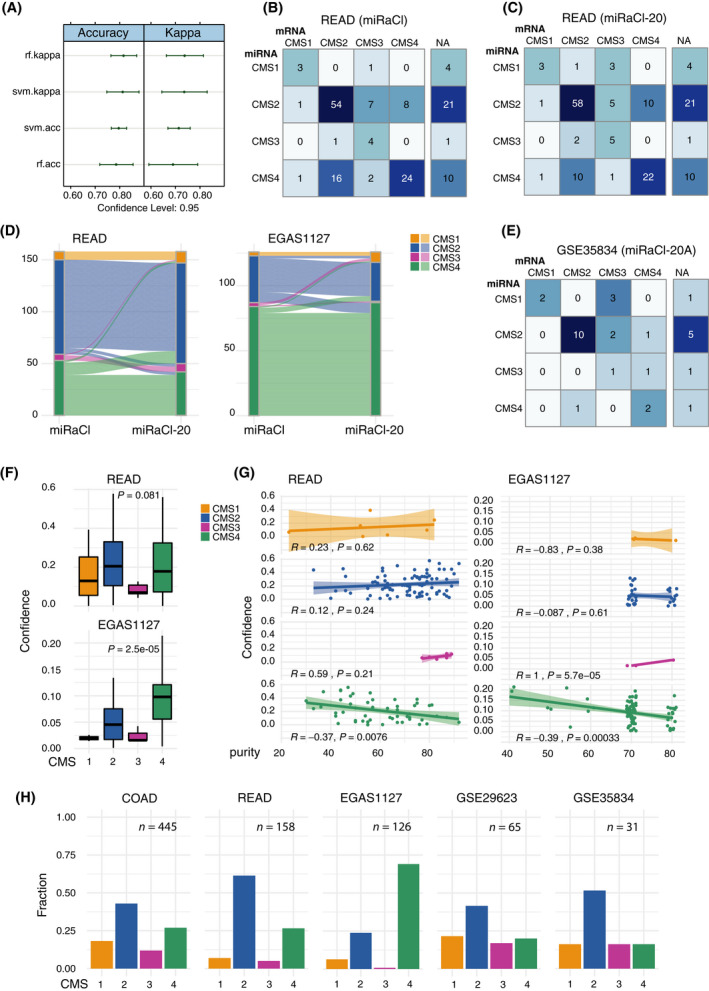
Performance of classifier. (A) Performance of different classifiers on the training dataset COAD (*n* = 271), random forest (rf), and support vector machines (SVM) optimizing Kappa or accuracy (acc), respectively. (B) Confusion matrix of CMS predictions from resulting miRNA‐assigned random forest CMS classifier (miRaCl) compared with mRNA‐based CMS classes using the rectal adenocarcinoma (READ) test dataset (*n* = 122). (C) Confusion matrix of CMS predictions from reduced random forest classifier based on the 20 most important features (miRaCl‐20) in rows compared with known mRNA‐based CMS classes in columns using the READ test dataset (*n* = 122). (D) The alluvial plot of miRaCl predictions from all 381 input features in comparison with the predictions of miRaCl‐20 in READ (*n* = 158) and EGAS1127 primary tumor samples (*n* = 126). (E) Confusion matrix of microarray‐based GSE35834 test dataset comparing CMS predictions from miRNA‐assigned classifier based on the 20 most important features in rows with mRNA‐based CMS predictions in columns as far as available (*n* = 23). (F) Confidence of miRaCl predictions was determined as the difference between the probabilities of the first and the second most likely class in READ (*n* = 158) and in EGAS1127 primary tumor samples (*n* = 126) and the means differed (tendentially) between the CMS classes (Kruskal–Wallis test). Boxes mark the interquartile range (IQR), whiskers extend to the furthest value within 1.5*IQR (Tukey whiskers). (G) We tested for correlation with the tumor purity (Pearson correlation test). (H) miRNA‐based CMS predictions as fractions of primary samples in training and test datasets, including samples where mRNA‐based classification was not possible.

The most optimal results in the classifier training were obtained with the training of a random forest optimizing Kappa instead of a support vector machine (Fig. 2A). When we trained a random forest‐based miRNA‐assigned classifier (miRaCl) on all suitable 381 miRNAs repeatedly on 80% (*n* = 217) of the COAD samples with robust mRNA‐based CMS labels, we obtained an average accuracy of 76.7% on the unseen samples (*n* = 54). When the number of features was reduced to keep only the features with the highest Gini importance, we observed a slightly higher accuracy of 77.9% with 20 miRNAs retained (miRaCl‐20).

The performance of the final model was tested in the READ dataset. We observed that the accuracy of the CMS predictions was slightly higher for miRaCl‐20 (72%) than for miRaCl (70%). The 95% confidence intervals (CI) were 0.6329–0.7987 for miRaCl‐20 and 0.607–0.7767 for miRaCl. The balanced accuracy was at least 75% for CMS1, CMS2, and CMS4 for miRaCl‐20 (Fig. S2C). The individual predictions plotted as confusion matrices showed that the majority of miRaCl (Fig. 2B) and miRaCl‐20 (Fig. 2C) predictions match the mRNA‐based CMS classes. Specifically, CMS3 was often mislabelled as CMS2, and CMS2 and CMS4 labels were swapped in a minor fraction of the samples. Discordance between miRaCl and miRaCl‐20 was uncommonly observed, only in 19 out of 122 (15.6%) samples, (Fig. 2D) and when we examined the dataset EGAS1127 in 17 out of 126 (13.5%) samples. Moreover, we demonstrated in a microarray‐based dataset that 20 miRNAs are sufficient for the prediction of CMS classes with an accuracy of 65.2% (95% CI 0.4273, 0.8362) in an additional test set (Fig. 2E).

As a parameter to measure the confidence of the prediction, we compared (subtracted) the probabilities of the first and second most likely class prediction, with lower values reflecting a lower confidence. From this analysis, it was apparent that confidences in CMS3 and CMS1 predictions were lower in both datasets (Fig. 2F). A lower prevalence of CMS1 in the READ dataset was expected due to the preferential right‐sided location of CMS1 tumors [1]. The lower prevalence of CMS1 and CMS3 tumors in the metastatic dataset EGAS1127 was in accordance with their lower rate of metastases and their decreased fraction of stage IV CRC [44]. When excluding predictions with lower confidences (< 25th percentile), the accuracy in the READ dataset was improved (76% for miRaCl and 79% for miRaCl‐20), with no clear separation of clinical parameters as stage or histology (Fig. S2D). The confidence to predict CMS4 was reduced when the tumor purity was high (Fig. 2G); however, the confidence of CMS1 and CMS2 was not affected by the tumor purity. The confidence in CMS3 tended to correlate positively with tumor purity. These observations were similar in miRaCl and miRaCl‐20 (Fig. S2E,F). The distribution of CMS class predictions based on miRaCl‐20 showed variations between the datasets (Fig. 2H). This was expected due to varying clinical compositions of the datasets, i.e., rectal location in READ or metastatic disease in EGAS1127 (Fig. S1E).

When the performance of miRaCl was examined on COAD or READ samples that could not be classified based on mRNA, a good correlation of posterior probabilities between miRaCl‐ and mRNA‐based predictions by CMS classifier was observed (Fig. S3A,B). The comparison of the derived confidences revealed a low correlation (Fig. S3C,D). This suggests that the samples that were more difficult to classify were different between mRNA and miRNA‐based classification. The alluvial plot indicated that multiple samples classified differently between mRNA and miRNA‐based prediction (Fig. S3E,F). This result should be interpreted with caution since these samples were not robustly classified by the standard mRNA‐based method (CMS classifier) including *P*‐values > 0.05. By censoring labels for 25% of samples with the lowest miRaCl confidence, the overall accuracy in this comparison increased from 56.2% to 61.6% for the COAD test set samples (*n* = 169) and from 45.7% to 52% for the READ samples that had impossible or inconsistent mRNA‐based classification (*n* = 35). Altogether, the data indicate that many of the excluded samples remain difficult to classify with miRaCl.

To investigate the applicability of miRaCl to FFPE tissue, we used COAD sample pairs from fresh frozen and FFPE tissue (*n* = 7) and observed a very good correlation of posterior probabilities between these replicates (Fig. S3G). However, due to four out of the seven samples being classified as CMS1, the results obtained in this patient cohort are potentially not suitable for generalization. Furthermore, we found that one sample shifted from CMS2 to CMS4 in FFPE compared with fresh frozen samples.

### 3.3 Importance of miRaCl features

**Fig. 3:**
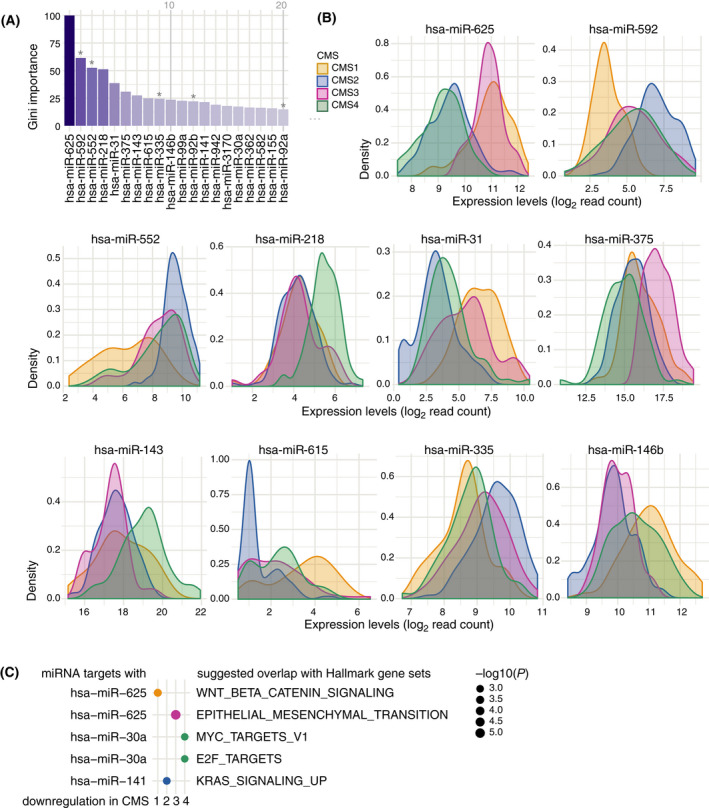
Important features of miRaCl. (A) Importance (Gini index) identified during the miRaCl training on colon adenocarcinoma dataset COAD, shown for the 20 features with the highest mean decrease in impurity. Asterix marks miRNAs previously reported as tumor‐specific [22]. (B) Density distributions (Gaussian kernel) of miRNA expression levels (read counts on log2 scale) in COAD stratified by known mRNA‐based CMS for the 10 most important miRNAs, which are used in miRaCl and miRaCl‐20. (C) Genes predicted to be targets of the miRNA from miRaCl‐20 were first intersected with genes downregulated in each CMS and afterwards tested for overlap with Hallmark gene sets. For each CMS, we show the miRNA with the lowest *P*‐value (one‐sided hypergeometric tests).

In order to understand which miRNAs the classifier is based on, we examined its most important features (Fig. 3A) obtained by the mean decrease in impurity (Gini importance) in more detail. Five of the 20 most important miRNAs have previously been reported to be significantly upregulated in CRC tumor tissue compared to surrounding normal tissue: miR‐592, miR‐552, miR‐335, miR‐92b, and miR‐92a [22].

In the density plots of the features with the highest Gini importance, which are relevant for both miRaCl and miRaCl‐20, we investigated the separation of the miRNA expression per class (Fig. 3B, Fig. S4A). For classification it is equally useful to know whether a feature is depleted or enriched in one or more classes, thus most features carry multiple information. For example, a low expression of miR‐625 makes it likely to be a CMS2 or CMS4 tumor and a high expression makes it likely to be a CMS1 or CMS3 tumor. A low expression of miR‐592 makes the tumor more likely to be of class CMS1 than CMS2 and vice versa.

A comparison of the most important features between miRaCl(‐20) and the microarray‐based adaptation miRaCl‐20A revealed that miR‐552, miR‐592, miR‐31, miR‐155, and miR‐625 were reproducibly important for the discrimination of CMS (Fig. S4B).

### 3.4 Regulatory role of miRaCl features

**Fig. 4:**
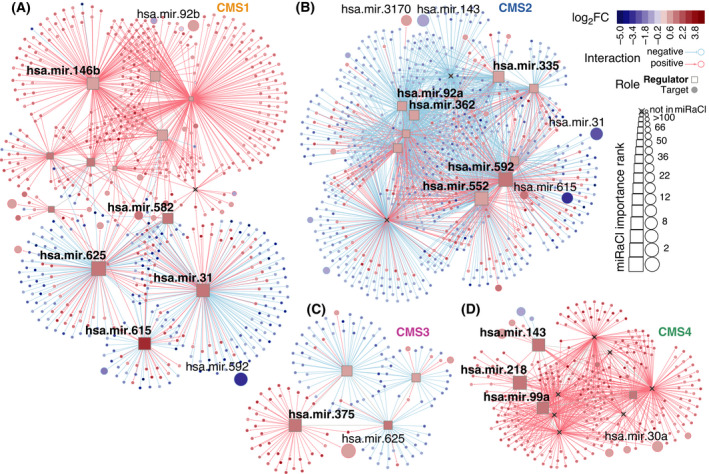
Features of miRaCl in regulatory networks. Regulatory networks were constructed from colon adenocarcinoma dataset COAD using up to 200 most differentially expressed mRNAs with absolute log2 fold change |log2FC| > 0.85 adjusted *P*‐value (*P*adj) < 0.001 per CMS and the most differentially expressed miRNAs with |log2FC| > 0.71 and *P*adj < 0.05 (Wald statistic, Benjamini–Hochberg corrected). Regulatory elements were identified amongst upregulated miRNAs with *P*adj < 0.001 in each CMS, respectively (A–D). Importance in miRaCl is indicated as node size, members of miRaCl‐20 are named in bold font for regulators and regular font for targets.

Next, we aimed to explore the regulatory role of miRaCl and miRaCl‐20 features for differences between CMSs. Therefore, we constructed regulatory networks from genes differentially expressed in each CMS and visualized the miRaCl feature importance in this context. To find regulatory roles among the most significantly upregulated (Fig. 4) or downregulated (Fig. S5) miRNAs in each CMS, we considered both mRNAs and miRNAs with differential expression in the same CMS as potential targets in the network analysis. Interestingly, among all significantly upregulated miRNAs with a regulatory role, more than one‐third (14/38) was also represented in miRaCl‐20, and this set is highlighted with the miRNA names displayed. In regard to the downregulated miRNAs with regulatory roles (15/44 in miRaCl‐20), we confirmed the importance of the miR‐200 family (miR‐141 and miR‐200c) in CMS4, as was previously reported [12].

By comparing the networks in both directions, we discovered a few overlaps with the potential to explain differences between the CMSs: miR‐92a, miR‐362, miR‐335, miR‐552, and miR‐592 were downregulated regulators in CMS1 and upregulated regulators in CMS2. We also found miR‐615 to have a diverging expression with regulatory roles in CMS1 and CMS2. Further examples were miR‐625 and miR‐99a with opposing expression between CMS1 and CMS4 and miR‐143 with downregulation in CMS2 and upregulation in CMS4.

To investigate relevant biological processes for the miRaCl‐20 features, we performed an overlap analysis between their predicted targets and the Hallmark gene sets (Fig. S4C) [43]. In order to refine the analysis and increase relevance for CMS, we narrowed down the set of predicted target mRNAs: Targets that were predicted by at least two databases were additionally intersected with mRNAs downregulated in each CMS to pin down the biological processes important for each CMS (Fig. 3C). Indeed, the identified pathways were in line with the known biology of the CMS, such as epithelial‐mesenchymal transition (EMT) being downregulated in the highly differentiated CMS3 and a downregulation of MYC targets in CMS4, as was previously described [1]. Furthermore, when a miRNA was suggested to target a pathway in a CMS, this miRNA was generally also highly expressed in the corresponding CMS (Fig. 3B, Fig. S3A).

### 3.5 Clinical implications

**Fig. 5:**
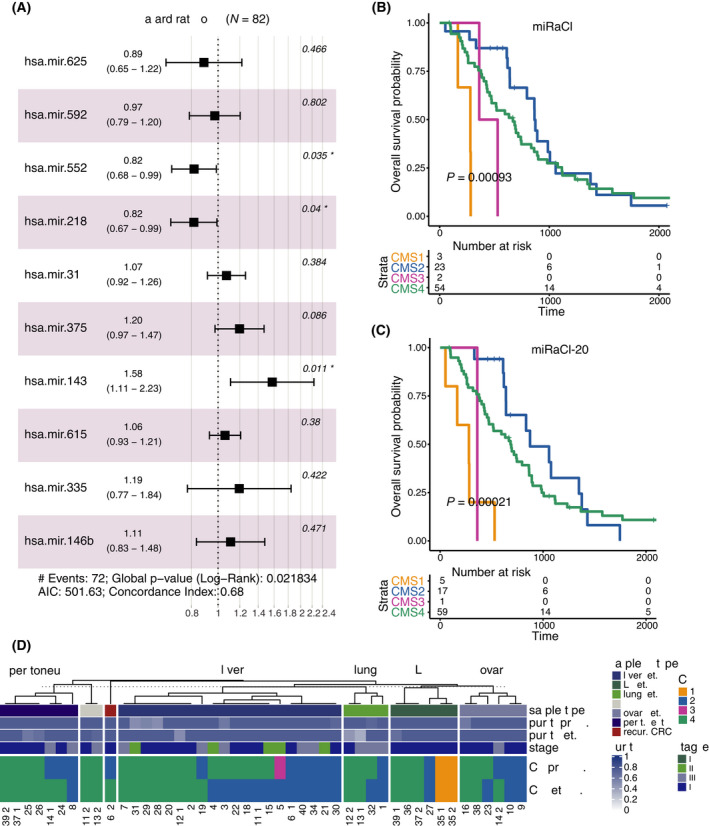
Clinical implications. (A) Cox proportional hazards model for the effect on overall survival (OS) for the 10 most important miRaCl/miRaCl‐20 features on EGAS1127 dataset, error bars represent 95% confidence intervals. (B, C) Kaplan–Meier analyses for OS stratified by the miRaCl or miRaCl‐20 predicted CMS classes in EGAS1127 dataset (*n* = 82), log‐rank *P*‐value from score test. (D) Comparison of miRaCl‐predicted CMS classes in patients with paired primary (prim.) and metastasis (met.) samples, including one recurrent (recur.) colorectal tumor; LN, lymph node; NOS, not otherwise specified. If multiple metastases were available, the primary was duplicated for visualization purposes, as marked by underscore extension of patient IDs (*n* = 38).

To test whether the identified miRNAs that separate CMS classes were also related to the OS in the EGAS1127 dataset, we computed the hazard ratios in a multivariate model of the first 10 features in miRaCl/miRaCl‐20 (Fig. 5A). A slightly decreased hazard (0.82, 95% CI 0.68–0.99) was observed in miR‐552, a miRNA with the highest expression in CMS2. Using the TCGA dataset as an additional dataset to study survival, miR‐552 was confirmed as a positive prognostic marker of good survival in patients with CRC in all stages [HR = 0.89 (0.79–1.0)], and even more pronounced within Stage IV CRC [HR = 0.65 (0.49–0.85)]. For two miRNAs—miR‐218 and miR‐143—showing the highest expression in CMS4, we observed opposing hazard ratios on the OS in the EGAS1127 dataset (stage IV): a decreased hazard for miR‐218 (0.82, 95% CI 0.67–0.99) and an increased hazard for miR‐143 (1.58, 95% CI 1.11–2.23). This result was not confirmed in the TCGA COAD/READ datasets across all stages.

Of note, the OS based on miRaCl(‐20)‐CMS predictions was worst for CMS1 (Fig. 5B,C), as previously reported for metastatic CRC [45, 46], although the number of cases predicted as CMS1 was low. As described in the original publication, CMS4 has a worse prognosis than CMS2 [1]. The difference between CMS2 and CMS4 is less pronounced in this metastatic cancer cohort than in the original study, which focused on earlier stages, in line with previous findings [45]. We confirm an enrichment of CMS4 and depletion of CMS1 (Fig. 2H) in this cohort consisting largely of advanced disease stages as described earlier [44].

In the EGAS1127 dataset, we retrieved CMS labels for the available metastatic samples from 38 patients and compared them with their corresponding primary samples. Though the CMS classification was not developed for use on metastases, we wanted to explore the concordance of CMS class predictions between primary and metastases pairs (Fig. 5D). We observed that peritoneal metastases were mostly classified as CMS4, whereas the liver metastases were enriched in CMS2 (*P*‐value 0.0355, Fisher's exact test).

## 4 Discussion

### 4.1 Training and validation of miRaCl

It has been widely acknowledged that molecular intertumor heterogeneity of CRC plays a major role in the clinical outcome of the disease, and this notion has resulted in the development of CMSs [1]. The typical CMS classification is dependent on the availability of mRNA expression data. To allow for the identification of CMSs based on an additional source of molecular information, we generated a miRNA‐assigned CMS classifier, CMS‐miRaCl. The parsimonious version of this classifier could predict unseen samples with an average accuracy of 77.9% within the COAD training dataset. In comparison, imCMS, the image‐based classifier, which made use of histochemical staining, had an accuracy of 70% within the training dataset [9]. We investigated the accuracy of miRaCl(‐20) and the correlation with clinical characteristics in two additional, completely independent datasets. While there was a good concordance of CMS1 between mRNA‐ and miRaCl‐based predictions, CMS3 seemed to be relatively frequently misclassified. Often, mRNA‐based CMS3 predictions were misclassified by miRaCl as CMS2, indicating that the distinction between the two classes could be challenging. One strategy to circumvent this problem is to combine CMS3 samples together with CMS2 samples into an “epithelial‐like” group, which was implemented by imCMS as well [11]. Overall, miRaCl(‐20) showed a good accuracy in the READ test set. Especially, by censoring samples within the lower quartile of confidence, the accuracy increased from 72% to 79% in miRaCl‐20 and from 70% to 76% in miRaCl. This opens the opportunity to choose the higher accuracy over the completeness of predictions. The parsimonious miRaCl‐20 was slightly more accurate than miRaCl. This might indicate that the lower number of features in miRaCl‐20 helps to avoid overfitting and supports generalizability [47].

Reduced accuracy and incongruences between mRNA and miRNA predictions might be in part related to intratumor heterogeneity. For example, mRNA‐based CMS class predictions can even differ between the tumor center and the invasive front in samples from the same tumor [48]. Although all TCGA mRNA and miRNA data pairs were retrieved from the same sample and vial, the portion of the sample used for the analysis was not the same for all cases. This could be one explanation why approximately one in four cases of both CMS2 and CMS4 were swapped by miRaCl(‐20) in comparison with the mRNA classification in the READ test set, even though they are considered to be quite different from each other. To identify the role of tissue composition, we tested whether the confidence of prediction was related to tumor purity and found an inverse correlation in CMS4 but no correlation in CMS2. Since miRNAs are more tissue‐specific than mRNAs [16], the inverse correlation between confidence and tumor purity in CMS4 is not surprising; if the defining mesenchymal component of this subtype is reduced, the sample becomes more difficult to classify. As another reason for misclassification related to tissue specificity of miRNAs, we consider the tumor location (left vs. right). Because multiple miRNAs are differentially expressed between the tissue of the colon and rectum [49], the location‐specific expression of miRNAs might lead to a reduced accuracy of miRaCl(‐20) in the READ dataset after training on the COAD dataset. For example, miR‐155 is included in miRaCl‐20(A) and is higher expressed in rectal cancer than in colon cancer samples [50]. Thus, it is one example of miRNAs that might influence the performance of the classifier depending on the location of the tumor. Furthermore, since CMS2 and CMS3 tumors consist mainly of epithelial cells, these classes might be especially sensitive to the specific tissue of origin.

The tissue specificity of miRNAs might also be relevant when the miRaCl(‐20) classifier is applied to metastatic samples. Contamination with noncolon tissue in metastases might influence the classifier, and thus, the prediction of metastases samples should be interpreted with caution. Surprisingly, the concordance of miRNA‐based CMS predictions between the paired primary and metastases samples seems to be higher than in previous reports, where mRNA‐based CMS predictions mostly differed between the primary tumor center and the lymph node metastasis [48]. This observation is in line with the previous observation that miRNA expression levels are highly correlated between paired metastases and primary samples [22]. However, it is important to note that neither the mRNA‐based CMS classifier nor miRaCl(‐20) was developed to be used on metastases.

The classification result could be influenced by the (clinical) composition of the classified cohort, as has been discussed for the mRNA‐based classifier [51]. Therefore, we tested whether the classification of samples was sensitive to the cohort composition in the EGAS1127 dataset. The CMS classification of primary tumor samples on the EGAS1127 dataset remained consistent, regardless of primary tumor and metastases samples (*n* = 172) were jointly classified using miRaCl or the primary samples were classified separately (*n* = 126) (data not shown). The ability to predict CMS from 20 miRNAs in microarray data was also tested and gave correct CMS classifications with an accuracy of about 65% in the dataset GSE35834. However, we did not test the classifier on other dataset compositions with different clinical characteristics such as a dataset consisting mainly of early‐stage colon cancer samples. To prove the consistency throughout all CRC tumor stages, it would be interesting to study the performance of miRaCl‐20 on additional datasets.

### 4.2 Role of important miRaCl(‐20) features in CMSs

Due to a redundancy of gene regulation by miRNAs or correlating expression of miRNAs from the same family, gene signatures for the same phenotype can differ depending on the analysis [52]. However, miR‐625, miR‐592, miR‐552, miR‐31, and miR‐155 were reproducibly important for the discrimination of CMSs as we confirmed with the microarray version miRaCl‐20A. Therefore, we reflected upon their potential biological roles in CMSs. The most important feature of miRaCl(‐20), miR‐625, was significantly upregulated in CMS1 and CMS3 and significantly downregulated in CMS2 and CMS4. Interestingly, a recent single‐cell study described that CMS1 and CMS3 share some similarities in their epithelial cell compartment [53]. Furthermore, miR‐625 was previously identified to be associated with MSI [54]. The pathway analysis suggested that miR‐625 might be involved in the differential regulation of Wnt signaling between CMS: It has predicted targets among the Wnt signaling pathway (*AXIN2*, *NKD1*), which were highly downregulated in CMS1 in comparison with other CMSs.

As miRNA with the potential to discriminate CMS2, we identified miR‐592 and miR‐552 among the most important miRaCl(‐20(A)) features. The presented network analyses suggested that miR‐592 has a regulatory role among the upregulated miRNAs in CMS2 and among the downregulated miRNAs in CMS1. Interestingly, miR‐592 has previously been linked to both tumor suppressive and tumor promotive characteristics in different cancer types [55, 56, 57, 58, 59, 60]. Of note, miR‐592 was the most differentially expressed miRNA between tumors of patients with a clinical benefit versus progressive disease on first‐line systemic treatment in advanced CRC [23]. Based on the pathway analysis we performed, we can speculate that the target genes of miR‐592 could be related to the EMT pathway. More is known about the mechanism of miR‐552: It has been found to be upregulated in CRC, compared with normal colon tissue, and it plays a role in promoting cell proliferation and migration *in vitro* [22, 61]. Moreover, miR‐552 appears to be a direct target of Wnt signaling and in turn targets TP53 [62], inducing Wnt/β‐catenin signaling [61], consistent with the presence of Wnt signaling in CMS2 [1]. In different datasets, EGAS1127 and TCGA COAD and READ, we found miR‐552 to have a significantly decreased hazard ratio for the OS. It seems interesting to further explore the relevance of miR‐552 as a prognostic biomarker.

The miRNA miR‐31 was upregulated in CMS1 and is known to be an established immunomodulatory miRNA, which is deregulated in autoimmune disorders [63]. In CRC, miR‐31 was previously found to be associated with worse differentiation [64] and immune infiltration [65], coinciding with CMS1 characteristics [1, 66].

Overall, several promising findings regarding the roles of miRNAs in CMSs suggest that the identification of novel molecular subtypes from miRNA data might further elucidate intertumor heterogeneity and could be an interesting subject for future research.

## 5 Conclusion

We developed a random forest classifier to separate CMSs based on miRNA expression. The parsimonious version miRaCl‐20 is able to determine the CMS in unrelated datasets with an average accuracy of > 70% across all classes based on only 20 miRNAs in the largest dataset. When the less represented group CMS3 or low confidence predictions are disregarded, the accuracy rises to > 75%. Additionally, the prediction of CMS4 appears to be influenced by tumor purity. During the classifier training, the importance of miRNAs was ranked. This provided insight into regulatory mechanisms potentially underlying the differences between CMSs. In highlight, miR‐552 is an interesting candidate for further evaluation as a prognostic biomarker. The application of miRaCl in an independent dataset of metastasized CRC allowed us to recapitulate the prognostic value of CMS classification.

## Conflict of interest

LV is a New York Stem Cell Foundation—Robertson Investigator. FM is the founder, director, and shareholder of Tailor Bio.

### Peer review

The peer review history for this article is available at https://publons.com/publon/10.1002/1878‐0261.13210.

## Author contributions

RSA, FM, HMWV, TEB, and LV conceptualized the project. RSA, LFM, XW, AT, TEB, and LV designed the analyses. RSA, DP, TdB, AT, and TEB acquired and curated samples and data. RSA and JS performed the analyses. RSA, LFM, XW, StH, PMGB, FM, TEB, and LV interpreted the results. RSA and LV wrote the manuscript. All authors were involved in critical revision and approval of the final manuscript.

## Supporting information


**Fig. S1.** Supplementary dataset description.
**Fig. S2.** Supplementary performance of classifier.
**Fig. S3.** Supplementary test set examination for miRaCl.
**Fig. S4.** Supplementary features of miRaCl‐20.
**Fig. S5.** Features of miRaCl in regulatory networks.

**Table S1.** Predicted miRNA targets overlapping downregulated genes in CMS.

## Data Availability

Scripts to reproduce miRaCl(‐20) can be found on https://github.com/rsmadam/CMS‐miRaCl.
