# Supplementary material for: Development of a miRNA‐based classifier for detection of colorectal cancer molecular subtypes
Source: Mol Oncol. 2022 Apr 29;16(14):2693–709. doi: 10.1002/1878-0261.13210 (PMC9297751; doi:10.1002/1878-0261.13210)
Supplement: Supplementary file 1 — Fig. S1. Supplementary dataset description. Fig. S2. Supplementary performance of classifier. Fig. S3. Supplementary test set examination for miRaCl. Fig. S4. Supplementary features of miRaCl‐20. Fig. S5. Features of miRaCl in regulatory networks. [file MOL2-16-2693-s002.pdf]

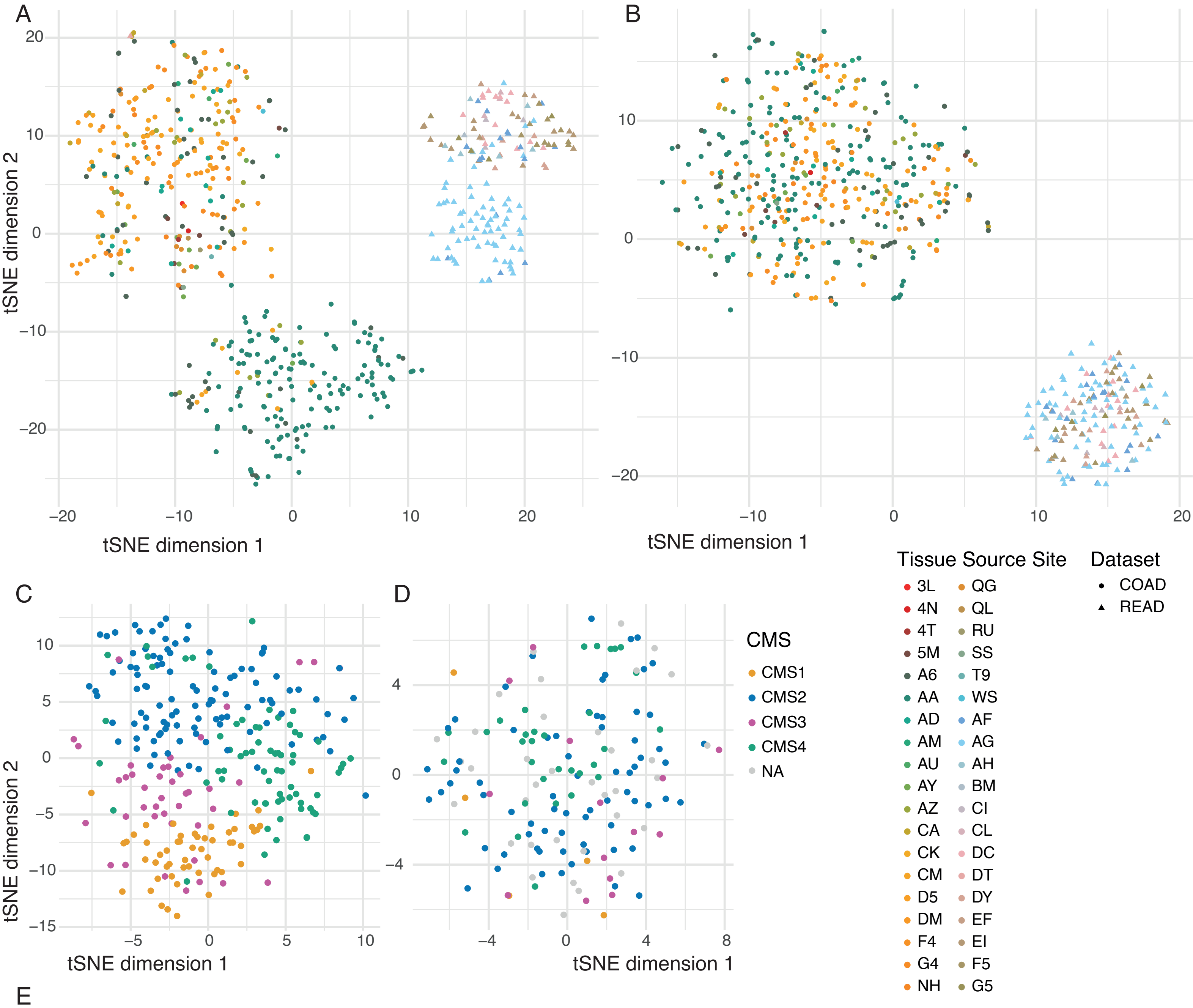

E

Characteristic		COAD (tr.) n = 271	COAD (test) n = 169	READ n = 158	EGAS1127 n = 126	GSE29623 n = 65	GSE35834 n = 31
**Gender**	Female (%)	43.9	53.3	46.2	39.0	38.5	25.8
	Male (%)	56.1	45.6	53.2	60.0	61.5	74.2
**Age**	Median (years)	68.6	69.4	66.2	65.0		62.0
	Mean	67.7	67.1	65.1	64.5	65	62.4
**Stage**	Stage I (%)	16.2	16.6	17.1	4.0	10.8	
	Stage II (%)	39.1	36.7	30.4	13.5	33.8	
	Stage III (%)	27.3	30.0	31.6	28.6	27.7	
	Stage IV (%)	15.1	13.6	14.6	52.4	27.7	
**CMS**	CMS1 (%)	19.6	19.5	3.2		18.5	6.5
	CMS2 (%)	39.5	34.3	44.9		30.8	35.5
	CMS3 (%)	15.5	20.1	8.9		14.4	19.4
	CMS4 (%)	25.5	26.0	20.2		16.9	12.9
	Not classifiable (%)	0.0	100.0	22.8		18.5	25.8

Figure S1 – Supplementary dataset description. (A) The miRNA datasets COAD and READ are clearly separated after individually normalizing the datasets using variance stabilizing transformation. The tSNE moreover gives indications for batch effects related to the Tissue Source Sites (TSS-codes in sample names). (B) After batch effect removal, the training and validation datasets (COAD and READ miRNA data) stay clearly distinct. (C) Class separation in tSNE for the training miRNA dataset from COAD based on CMS labels obtained from mRNA. (D) Class separation in tSNE for the validation miRNA dataset from READ based on CMS labels obtained from mRNA. (E) Table summarizing clinical characteristics of training (tr.) and validation datasets.

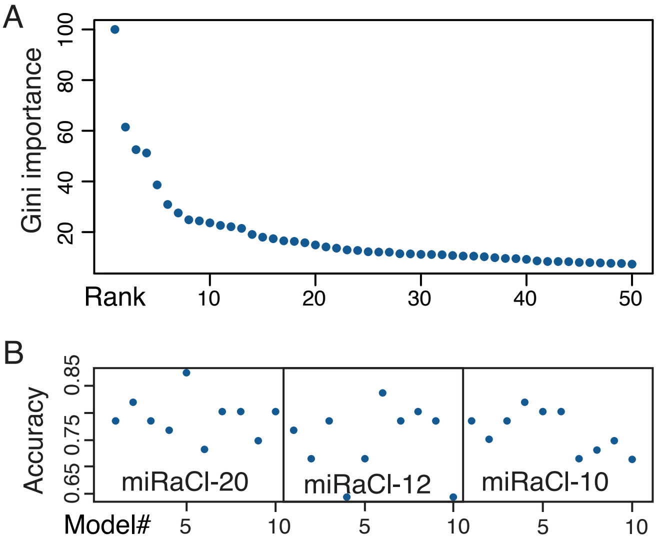

C

		Reference CMS (n)			
		CMS1 (5)	CMS2 (71)	CMS3 (14)	CMS4 (32)
miRaCl	Sensitivity	0.6000	0.7606	0.2857	0.7500
	Specificity	0.9915	0.6863	0.9907	0.7889
	Accuracy	0.7500	0.7714	0.8000	0.5581
	Balanced Accuracy	0.7957	0.7234	0.6382	0.7694
	Overall Accuracy	0.6967 (95% CI : 0.607, 0.7767)			
miRaCl-20	Sensitivity	0.6000	0.8169	0.3571	0.6875
	Specificity	0.9658	0.6863	0.9815	0.8667
	Accuracy	0.4286	0.7838	0.7143	0.6471
	Balanced Accuracy	0.7829	0.7516	0.6693	0.7771
	Overall Accuracy	0.7213 (95% CI : 0.6329, 0.7987)			

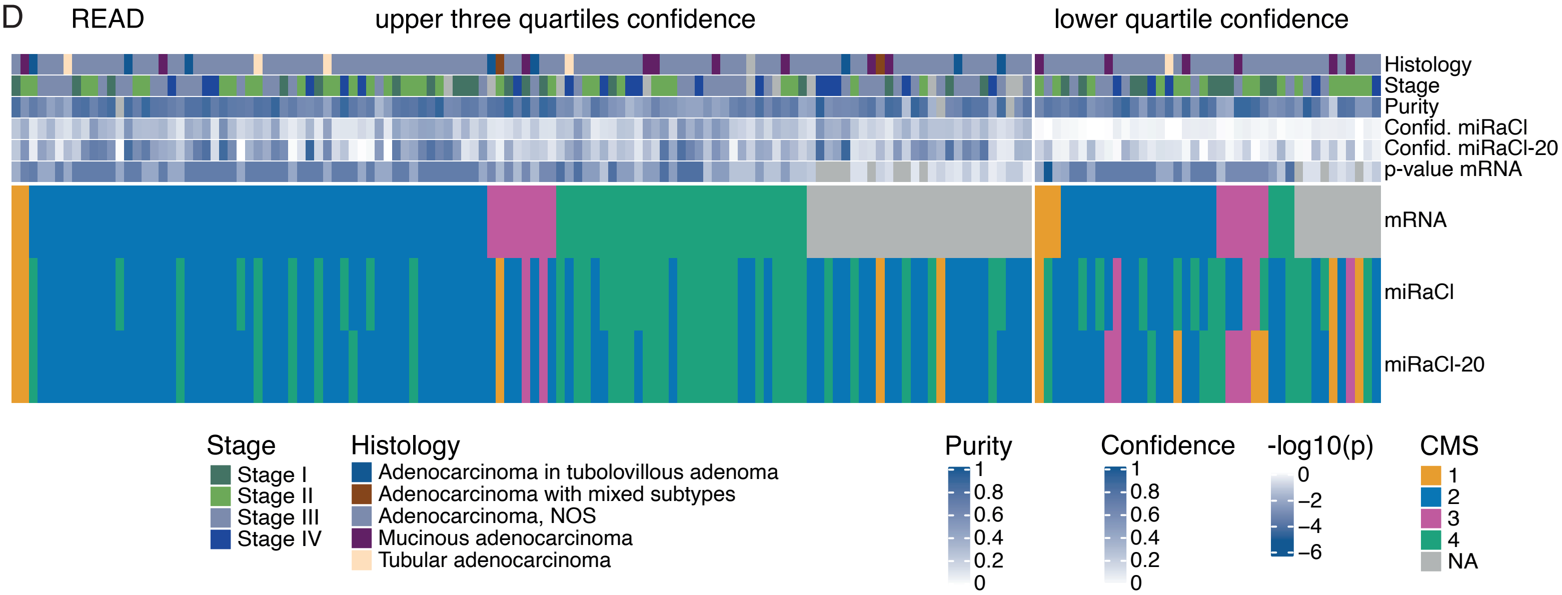

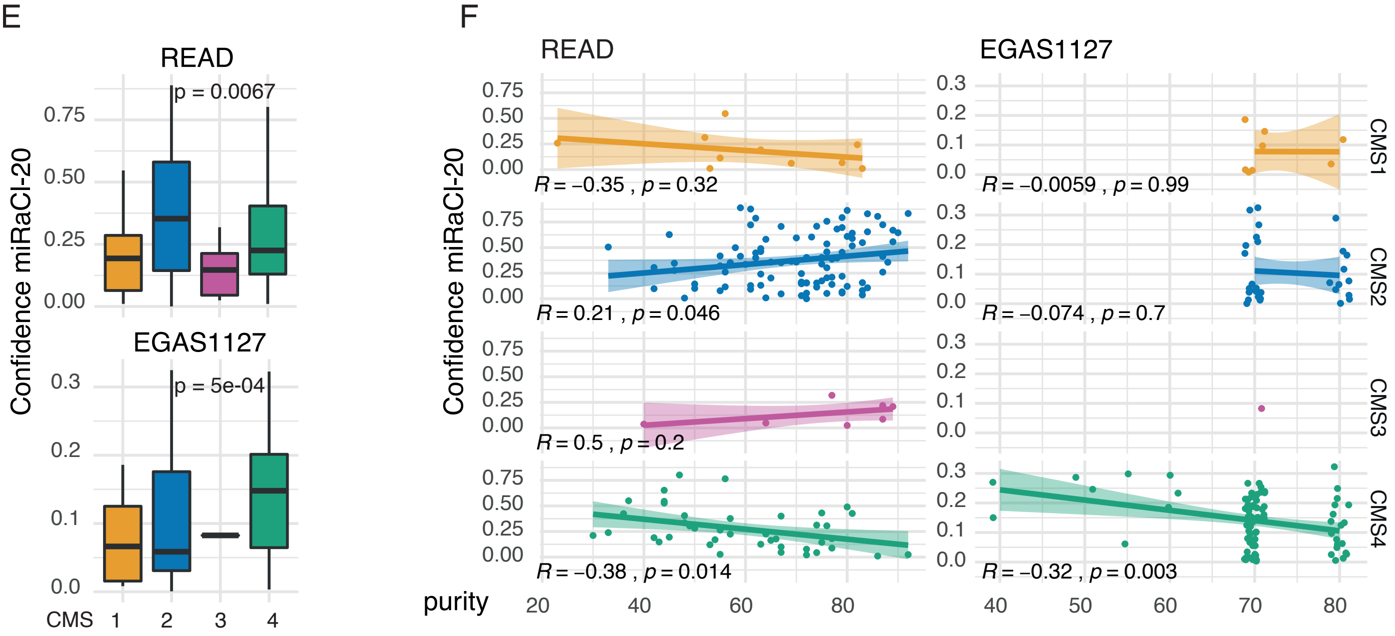

Figure S2 – Supplementary performance of classifier. (A) When we examined the mean Gini importance across 80 random forest classifier trainings we observed a steep decrease within the 10 most important features and a minimal decrease after the 20th rank. (B) When we tested to use only the 10 or 12 most important features for a mini-classifier, the accuracy tended to decrease compared to the miRaCl-20. (C) Performance measures of miRaCl and miRaCl-20 by class. (D) Heatmap annotating clinical parameters with the predictions of miRaCl and miRaCl-20 in rectal adenocarcinoma dataset READ, including samples which had no mRNA-based CMS prediction (n = 158). (E) Confidence of miRaCl-20 predictions was determined as the difference between the probabilities of the first and the second most likely class in READ (n = 158) and in EGAS1127 primary tumour samples (n = 126) and the means differed (tendentially) between the CMS classes (Kruskal-Wallis-Test). Boxes mark the inter-quartile range (IQR), whiskers extend to the furthest value within 1.5*IQR (Tukey whiskers). (F) We tested for correlation to the tumour purity (Pearson correlation test).

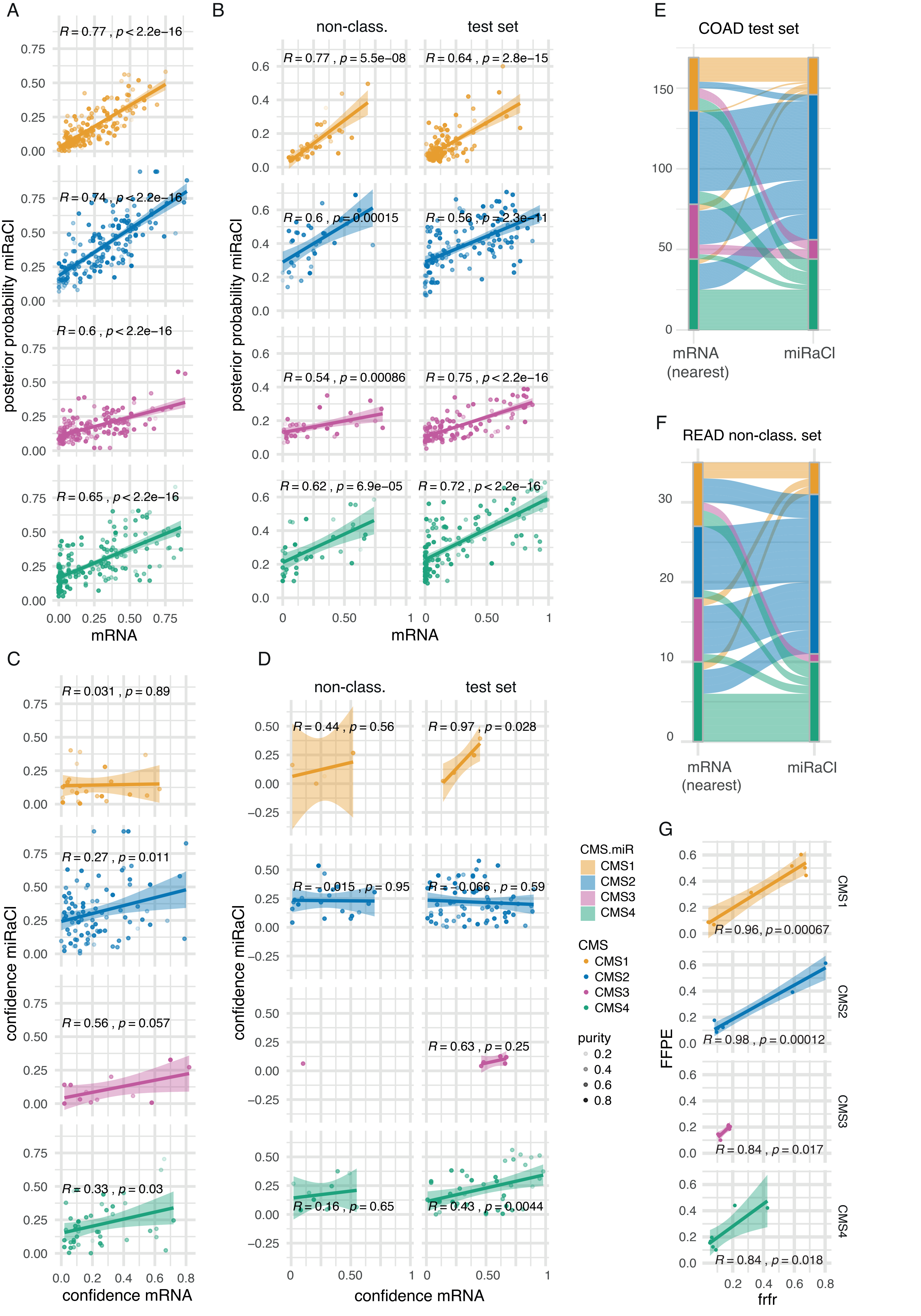

Figure S3 – Supplementary test set examination for miRaCl. For samples which had impossible/inconsistent mRNA-based classification (non-class.) from COAD (A; n = 168) and READ (B; n = 35), we retrieved the posterior probabilities from CMSclassifier (mRNA-based) and miRaCl (miRNA-based), as well as for the READ samples from the original test set (n = 122). We calculated the confidences of the most likely class from the difference between the first and second highest posterior probabilities from CMSclassifier and miRaCl, respectively, for COAD (C; n = 168) and READ (D; n = 35 and n = 122). The nearest CMS as predicted by the CMSclassifier, was compared to the miRaCl class prediction (E-F). (G) Correlation of the posterior probabilities from miRaCl for fresh frozen (frfr) COAD samples (n = 7) with their paired formalin-fixed paraffin-embedded tissue (FFPE) sample. All test statistics are referring to Pearson correlation testing.

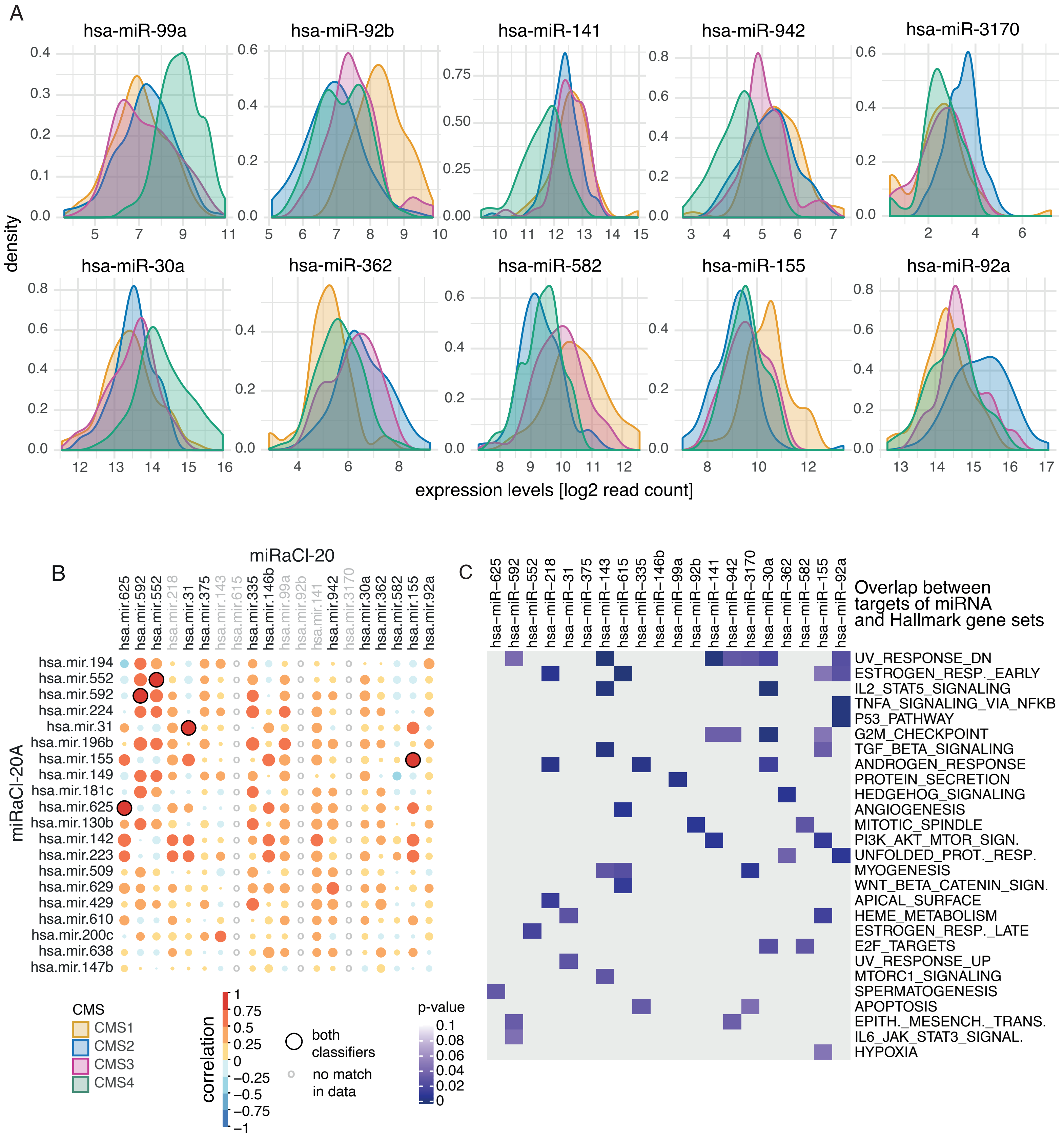

Figure S4 – Supplementary features of miRaCl-20. (A) Density distributions (Gaussian kernel) of miRNA expression levels (read counts on log2 scale) in COAD stratified by known mRNA-based CMS for the miRNAs with Gini importance rank 11-20, which are used in miRaCl and miRaCl-20. (B) In the microarray-based dataset GSE29623 we calculated and plotted the Pearson correlation between the 20 most important features according to the training in this dataset (rows) and the original 20 most important miRaCl-20 features (columns). Features of miRaCl in grey font were not found in GSE29623 or ignored before training due the removal of most highly correlated features within GSE29623. (C) Genes predicted to be miRNA targets were tested for overlap with Hallmark gene sets. Potential overlaps with p-values < 0.1 (one-sided hypergeometric tests) are shown in purple. Epith.=Epithelial, Mesench.=Mesenchymal, Prot.=Protein, Resp.=Response, Sign.=Signalling, Trans.=Transition.

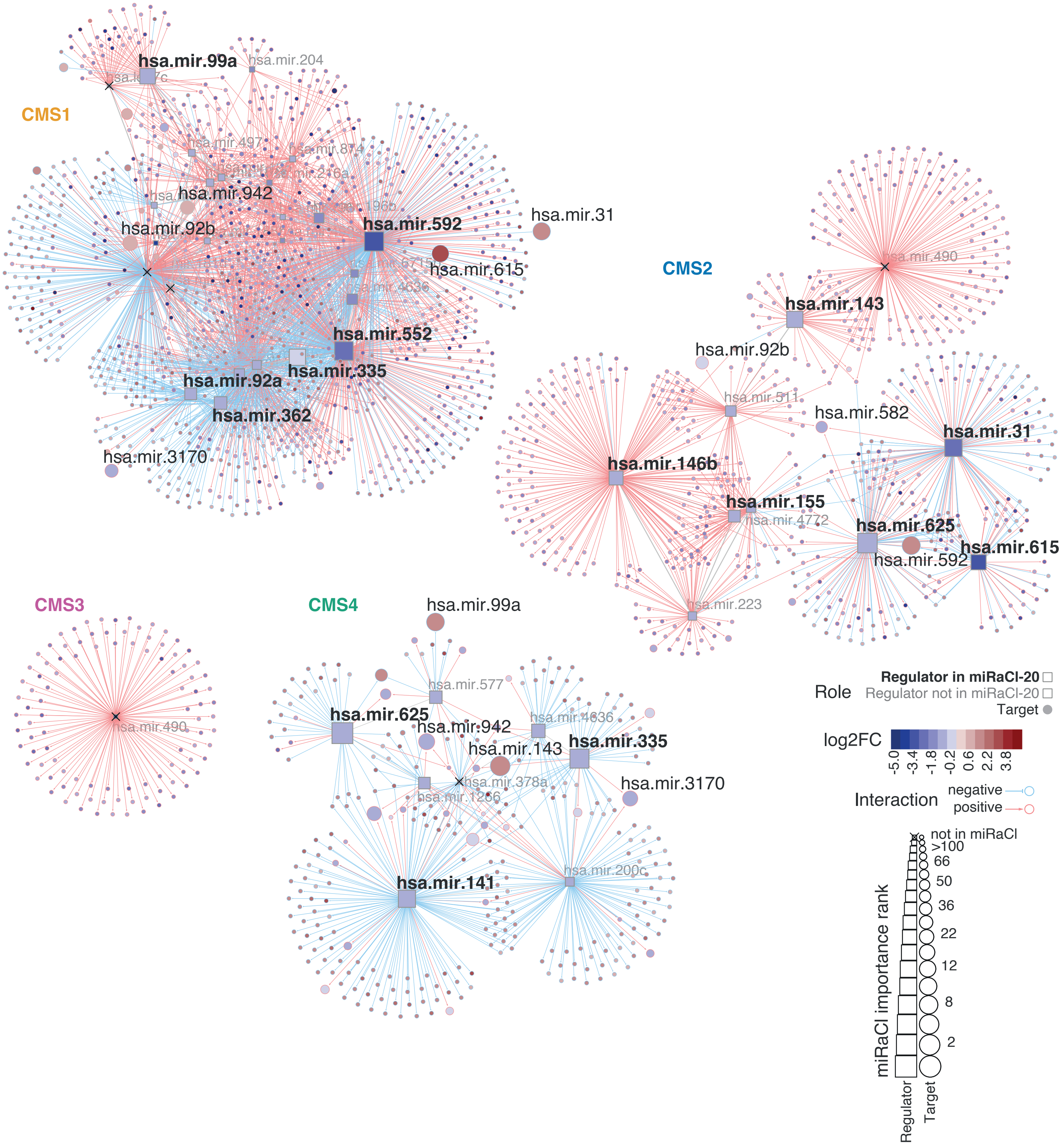

Figure S5 – Features of miRaCl in regulatory networks. (A) Regulatory networks were constructed from colon adenocarcinoma dataset COAD using up to 200 most differentially expressed mRNAs with absolute log2 fold change |log2FC| > 0.85 adjusted p-value (padj) < 0.001 per CMS and the most differentially expressed miRNAs with |log2FC| > 0.71 and padj < 0.05 (Wald-statistic, Benjamini-Hochberg corrected) as potential targets. Regulatory elements were identified amongst downregulated miRNAs with padj < 0.001 in each CMS respectively. Interactions with a negative co-expression suggesting a suppressive function are indicated by blue edges, interactions with a positive co-expression suggesting an activating function are indicated by red edges. Importance in miRaCl is indicated as node size, indicated miRNA names are black for miRaCl-20 members (bold for regulators, regular for targets) and grey for regulators not in miRaCl-20.
